# Prevalence, incidence, and re-occurrence risk of musculoskeletal pain in older adults in the United Kingdom: a population-based study

**DOI:** 10.3389/fpain.2023.1197810

**Published:** 2023-09-01

**Authors:** Maja R. Radojčić, Romain S. Perera, Deborah J. Hart, Tim D. Spector, Nigel K. Arden

**Affiliations:** ^1^ Nuffield Department of Orthopaedics, Rheumatology and Musculoskeletal Sciences, University of Oxford, Oxford, United Kingdom; ^2^ Division of Psychology and Mental Health, Faculty of Biology, Medicine and Health, University of Manchester, Manchester, United Kingdom; ^3^ Department of Allied Health Sciences, Faculty of Medicine, University of Colombo, Colombo, Sri Lanka; ^4^ Sports and Exercise Medicine Unit, Faculty of Medicine, University of Colombo, Colombo, Sri Lanka; ^5^ Department of Twin Research and Genetic Epidemiology, King’s College London, London, United Kingdom; ^6^ MRC Lifecourse Epidemiology Unit, University of Southampton, Southampton, United Kingdom

**Keywords:** prevalence, incidence rate, relative risk, re-occurrence, musculoskeletal pain, body mass index

## Abstract

**Background:**

Throughout the literature, pain burden has been assessed by asking different questions, often cross-sectionally, different populations of interest. We know little about pain re-occurrence and how to translate knowledge between pain questions within the population of interest. We aimed to estimate the burden of musculoskeletal pain by estimating prevalence, incidence rates, and re-occurrence risk of back, hand, hip, knee, and foot pain using different questions from UK population-based samples and predict the number of affected individuals in the UK in 2030.

**Methods:**

We used two UK population-representative studies, with two eight-year-apart follow-ups and two pain questions assessing recent pain episodes and often troubled pain when walking. We estimated prevalence, 8-year incidence rates, and 8-year pain re-occurrence risk for women and men aged 50 years and older and the relation between the two pain questions.

**Results:**

Among UK individuals older than 50 years, the prevalence of musculoskeletal pain episode was 20%–50%, and the incidence was 20–40/1,000 person-years, while the prevalence of pain when walking was 10%–25%, and the incidence was 6–12/1,000 person-years. The most prevalent musculoskeletal pain types were back and knee pain; of five women experiencing back or knee pain episodes, three are expected to be often troubled by pain. Hip and foot pain had similar estimates in both questions. Hand pain peaked in women aged 50–65 years. Women had higher prevalence and incidence rates, but men had higher 8-year re-occurrence risk of all types of musculoskeletal pain. Reporting a pain episode was associated with two times higher risk, but often troubled by pain when walking was associated with four to seven times times higher risk of the same pain in 8 years. Women and men with a body mass index (BMI) of ≥27 kg/m^2^ were twice as likely to experience musculoskeletal pain than those with BMI<27 kg/m^2^. In 2030, we expect 2–7 million people older than 50 years in the United Kingdom to seek site-specific musculoskeletal pain-focused healthcare.

**Conclusions:**

In individuals older than 50 years, the experience of musculoskeletal pain at least doubles the chance of experiencing it again. Women report musculoskeletal pain more often, but men report more persistent pain. Musculoskeletal pain presents a significant burden to public health.

## Introduction

Pain is “an unpleasant sensory and emotional experience associated with, or resembling that associated with, actual or potential tissue damage” (1). It is also the symptom that brings patients to seek medical care and, in chronic form, a health condition classified in the newest International Classification of Diseases (2). A 3-month duration is a criterion for defining chronicity. Chronic pain is a debilitating condition that negatively affects multiple aspects of individuals’ health and consequently attracts significant research attention (3, 4). In musculoskeletal disease, where early disease diagnosis and disease-modifying medications are lacking, pain is the primary patient complaint, the reason for visiting health professionals, and the treatment focus (5, 6). However, not every musculoskeletal pain is chronic. A pain episode can last several weeks, a month, or two, and not meet the chronicity criterion. Yet, these patients use healthcare: physician visits, diagnostic tests, pharmacological treatments, physiotherapy, or surgery. Besides pain presence, intensity, and chronicity, limited activities and reduced quality of life are important dimensions for understanding pain burden. Pain episodes can re-occur after some time, or the underlying disease can be manifested in flares, indicating a repeated need for pain-focused healthcare. Thus, having epidemiological estimates of pain episodes and a link between different questions that are used to assess pain properties is of great interest to public health (7).

Several reports provided prevalence estimates of musculoskeletal pain in specific occupational and general populations (4, 8, 9). A few UK reports also provided cross-sectional estimates based on different pain questions (10–12), but none of the musculoskeletal pain re-occurrence or incidence rates. The prevalence has been often reported per sex and age group, the recognised risk factors. However, the body mass index (BMI) is also a risk factor, but the estimates per BMI group are lacking (13–16).

Therefore, given the public health importance and knowledge gaps, we aimed to provide the prevalence, incidence, and risk of re-occurrence of musculoskeletal pain. We used two population-representative UK cohorts and two pain questions assessing pain episodes irrespective of the duration and often troubled by pain when walking as an essential determinant of quality of life (17). Also, we aimed to provide the national estimate for the near future—2030. Finally, we intended to provide estimates per sex, age, and BMI group, considering a recently proposed BMI cut-off for musculoskeletal pain (15).

## Methods

### Study samples

We used data from two English prospective population-based cohorts—the Chingford 1,000 women study and the English Longitudinal Study of Ageing (ELSA). The Chingford study started in 1989 in Chingford (Northeast London, UK) (18, 19). All women aged 45–65 years who registered in a large general practice were invited to partake in a longitudinal study assessing common medical conditions, particularly musculoskeletal diseases, and ageing. Of contacted, 1,003 (78% response rate) women participated in the first visit (18). The women have been followed up for 22 years, with irregular follow-ups, more frequent in the first decade (5, 14, 15). The Chingford study has been a UK women representative sample (19).

The ELSA study started in 2002 when women and men aged 50 years and older who participated in the Health Survey for England 1998/1999/2001 were invited for a longitudinal study focused on ageing (20). The study follow-ups (waves) were biannual interviews, with nurse visits every other wave starting from Wave 2, for clinical assessments such as body weight and height (13, 21). The study allowed the change in the interviewed household member over time, and new participants have been added over time. The ELSA study has been a UK household representative sample (21).

Here, we defined the study samples from the Chingford and the ELSA based on the following criteria: (1) available individual data on multi-site musculoskeletal pain on two assessments in longer term, i.e., closest to a decade apart and comparable between the studies, and (2) available data on age, sex, and body mass index. The data of interest were available approximately 8 years between the repeated assessments in both studies. Thus, we used the data from the follow-ups 1 and 8 from the Chingford and the Waves 2 and 6 from the ELSA performed in 1989, 1996, 2004, and 2012, respectively.

**Figure 1:**
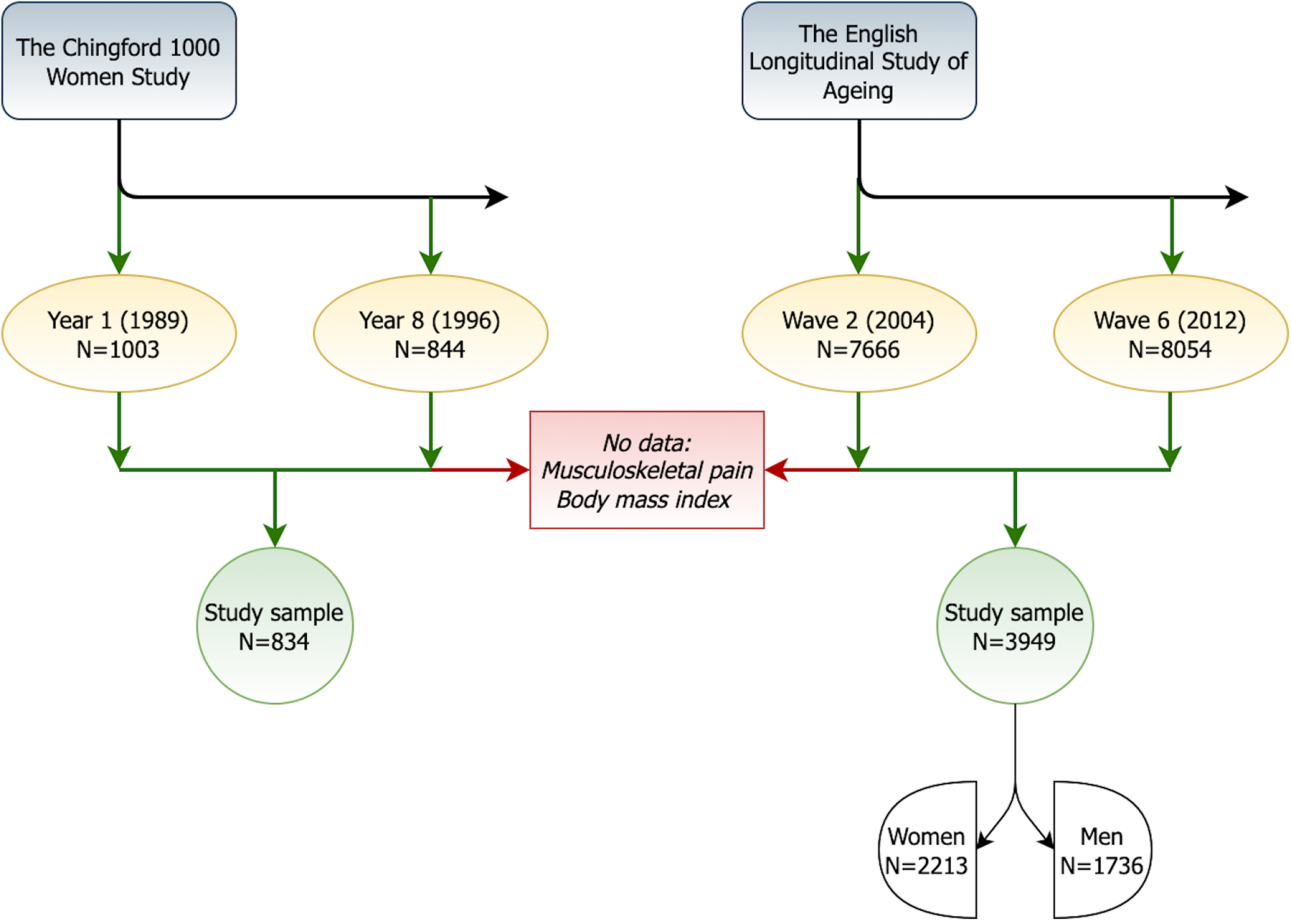
The study flowchart.

Of the 1,003 Chingford women who participated in the study in Year 1, 834 (83.2%) attended the Year 8 follow-up, had data on musculoskeletal pain and BMI, and made the Chingford study sample. The first nurse visit in the ELSA was at Wave 2 with 7,666 attending participants. Of these, 3,949 (51.5%) participated 8 years later at the Wave 6 nurse visit and had data on musculoskeletal pain and BMI. They formed the ELSA study sample (56% women). Figure 1 shows the study flowchart.

Finally, we used the UK demographic data from the Office for National Statistics (ONS). These include the population of women and men aged 50 years and older in 4 calendar years of the Chingford and ELSA assessments. Also, we used the ONS latest prediction (2020-based) for the UK population for 2030 (22).

The participants in both studies have provided their written informed consents for participation, and the relevant research ethics committees (for the Chingford: the Outer North East London Research Ethics Committee, and the ELSA: wave 2 - the London Multi-Centre Research Ethics Committee (MREC/04/2/006) and wave 6 - the NRES Committee South Central - Berkshire (11/SC/0374)) approved the studies.

### Musculoskeletal pain

The musculoskeletal pain assessment in the Chingford study focused on episodes. In Year 1, women were asked about back, knee, and hand pain. The back pain question was whether they ever experienced episodes lasting more than a week, while knee and hand pain assessed the current pain in the left or right knee or any interphalangeal joint, left or right (15). In Year 8, women reported whether, in the last year, they experienced pain episodes in the back, knee, hand, hip, and foot (14). All variables were binary and, given slight differences in formulated questions, were interpreted as recent pain episodes that women could recall at the assessment.

The pain assessment in the ELSA focused on walking/mobility as a determinant of quality of life (17, 23). Firstly, the participants reported whether they are often troubled by pain, and when they positively answered, they rated pain in their back, knee, hip, and foot when walking on a flat surface on a scale of 0 (no pain) to 10 (severe pain) (21). From this scale, we created binary variables where zero meant no pain and any other value was the presence of pain in the specific part when walking (13).

### Other measures

We used age, sex, and BMI for descriptive and stratification purposes (13, 15). All participants in the Chingford study were women. Their age was calculated as a difference between the assessment date and birth date, and their BMI from measured body weight and height (kg/m^2^) (15). The ELSA participants reported age and sex. During the nurse visit, the body weight and height were measured for BMI calculation (13). We created categorical age and BMI variables. Age categories were as follows: <50 (only in Chingford), 50–65, 65–80, and ≥80 years (only in ELSA). We used the BMI categories of the World Health Organization (WHO): underweight (<18.5 kg/m^2^), normal weight (18.5–25 kg/m^2^), overweight (25–30 kg/m^2^), and obese (≥30 kg/m^2^). In addition, we used a recently proposed BMI cut-off for musculoskeletal pain. A study reported that over 19 years, women with BMI of 25–27 kg/m^2^ had no different risk of musculoskeletal pain or mortality than women with BMI of <25 kg/m^2^ (15). Therefore, we dichotomised BMI into the <27 and ≥27 kg/m^2^ groups.

### Statistical analysis

We described the samples presenting means with standard deviation (SD) for continuous variables and frequencies (%) for categorical variables. We provided all sex-stratified estimates and visualised the prevalence of each pain type per age and BMI group. The prevalence of each musculoskeletal pain type was calculated as the number of cases that reported the pain by the total number of the included participants. We provided the pain prevalence at each assessment and both assessments. We computed the 8-year incidence rates by dividing the number of new cases at the second follow-up by the total number of person-years (expressed per 1,000 person-years). We provided 95% confidence intervals for the incidence rates using the Mid-P exact test implementing Miettinen's modification of the Fisher’s test (24). We computed the re-occurrence risk as the ratio of the musculoskeletal pain risk/probability at the second follow-up (outcome) among those who reported the same pain type at the first assessment (exposed) to the outcome probability among those who did not report the pain at the first follow-up (non-exposed) (25).

Further, we used the observed data from the two UK cohorts and the ONS-provided UK population data to predict the number of affected individuals by each pain type on the population level and link estimates from two questions to the UK population in 2004, 2012, and 2030 years. To calculate the number of UK women with pain episodes in 2004 and 2012, we used the prevalence estimates from 1996 because there were more pain sites available. The prevalence estimates of pain when walking from 2004 to 2012 were recalculated to the year population estimates. Finally, we used ONS 2020-based predictions for the UK population in 2030 (22) and calculated the predicted number of individuals affected by each pain type using the 1996 and 2004 prevalence estimates. We also expressed these as the number of cases per 1,000 individuals.

We performed additional analyses exploring the proposed BMI cut-off for musculoskeletal pain (27 kg/m^2^). We provided prevalence per group. Further, we computed the prevalence ratio for each pain type as the ratio of the musculoskeletal pain risk/probability (outcome) among those who had BMI of ≥27 kg/m^2^ (exposed) to the outcome probability among those who had BMI of <27 kg/m^2^ (non-exposed) at the same assessment.

We used SPSS Statistics 27 (IBM, Chicago, IL, USA) and Microsoft Excel (version 16) for statistical analyses and graphic presentations.

## Results

The descriptive statistics of the study samples are presented in Table 1. In Year 1, the Chingford women were, on average, 54 years old (SD = 5.9), with 29.6% younger than 50 years. They had an average BMI of 25.4 kg/m^2^ (SD = 4.1), which increased to 26.7 kg/m^2^ (SD = 4.7) in Year 8. In Wave 2, the participants of the ELSA were, on average, 64 years old (SD = 7.8), with 3.4% older than 80 years. Their average BMI was 27.9 kg/m^2^ (SD = 4.7) and similar 8 years later, which was 28.2 kg/m^2^ (SD = 5.1).

**Table 1:** Descriptive statistics of the study samples.

Variable/study assessment (year)	The Chingford study		The ELSA study					
	*N* = 834		*N* = 3,949					
	Year 1 (1989)	Year 8 (1996)	Wave 2 (2004)			Wave 6 (2012)		
	Women	Women	All	Women (56%)	Men (44%)	All	Women (56%)	Men (44%)
Age (years), mean (SD)	54.4 (5.9)	61.2 (5.9)	63.7 (7.8)	63.8 (7.9)	63.5 (7.7)	71.4 (7.7)	71.5 (7.8)	71.2 (7.6)
Age groups (years), %
<50	29.6	—	—	—	—	—	—	—
50–65	70.4	70.1	57.9	57.4	58.5	22.4	22.3	22.6
65–80	—	29.9	38.7	39.0	38.5	60.4	60.1	60.7
≥80	—	—	3.4	3.7	3.1	17.2	17.6	16.8
Body mass index, mean (SD)	25.4 (4.1)	26.7 (4.7)	27.9 (4.7)	28.0 (5.1)	27.8 (4.1)	28.2 (5.1)	28.3 (5.5)	28.1 (4.4)
Body mass index groups (kg/m^2^), %
<18.5	0.7	1.0	0.6	0.7	0.4	0.9	1.2	0.5
18.5–25.0	52.0	38.6	26.5	29.5	22.8	26.5	29.2	23.1
25.0–30.0	34.7	39.6	44.9	39.5	51.8	41.9	36.4	48.9
≥30.0	12.6	20.9	28.0	30.4	25.1	30.7	33.2	27.5

<27	71.8	58.8	47.3	48.4	45.9	44.7	45.5	43.7
≥27	28.2	41.2	52.7	51.6	54.1	55.3	54.5	56.3

Back pain was the most prevalent type considering either question, followed by knee pain (Table 2). The 8-year re-occurrence risk of back pain episodes was 1.80 (95% CI 1.58–2.02), and of often troubled pain when walking was 4.23 (95% CI 4.09–4.38) for women and 5.34 (95% CI 5.14–5.55) for men. We found that in 2030 in the United Kingdom, we can expect 5.8 million women affected by back pain episodes, and 3.4 million women and 2 million men often troubled by activity-limiting back pain. Thus, out of five women experiencing a back pain episode, three are expected to be often troubled by back pain. We found very similar estimates for knee pain. Hand pain episodes had the highest incidence rate. The prevalence of hip and foot pain when walking was lower than the same type of knee pain, and the highest re-occurrence risk of musculoskeletal pain had men troubled by hip and foot pain. In 2030, we expect that out of four women experiencing a hip pain episode, three will be often troubled by hip pain, and out of five women reporting a foot pain episode, four women will be often troubled by it.

**Table 2:** Musculoskeletal pain in individuals aged 50 years and older in the United Kingdom.

	Sample estimates					Predicted UK population estimates			
Pain variable	Prevalence, *N* (%)			Re-occurrence risk ratio (95% CI)^a^	Incidence rate/1,000 person-years (95% CI)^b^	Prevalence, *N*^c^		Prevalence, *N*/1,000^d^	
Pain episode^e^	1989	1996	Both years			2004	2012	2030	2030
Back pain									
Women	308 (52.5)	233 (39.7)	155 (26.4)	1.80 (1.58–2.02)	18.98 (15.11–23.56)	4,307,258	4,704,549	5,788,721	397
Knee pain									
Women	189 (32.2)	240 (40.9)	115 (19.6)	1.94 (1.75–2.12)	30.42 (25.43–36.12)	4,436,661	4,845,887	5,962,631	409
Hand pain									
Women	203 (34.6)	303 (51.6)	135 (23.0)	1.52 (1.37–1.67)	40.88 (35.04–47.43)	5,601,284	6,117,932	7,527,821	516
Hip pain									
Women	—	156 (26.6)	—	—	—	2,883,830	3,149,827	3,875,710	266
Foot pain									
Women	—	135 (23.0)	—	—	—	2,495,622	2,725,811	3,353,980	230
Pain when walking	2004	2012	Both years			2004	2012	2030	2030
Back pain									
Women	517 (23.4)	520 (23.5)	293 (13.2)	4.23 (4.09–4.38)	12.82 (11.23–14.57)	2,535,083	2,784,980	3,407,014	234
Men	265 (15.3)	259 (14.9)	127 (7.3)	5.34 (5.14–5.55)	9.50 (7.98–11.23)	1,419,067	1,571,477	2,011,780	153
Knee pain									
Women	504 (22.8)	473 (21.4)	269 (12.2)	4.47 (4.32–4.62)	11.52 (10.02–13.19)	2,471,338	2,533,261	3,321,344	228
Men	285 (16.4)	251 (14.5)	128 (7.4)	5.30 (5.09–5.51)	8.86 (7.39–10.53)	1,526,166	1,522,938	2,163,613	164
Hip pain									
Women	416 (18.8)	349 (15.8)	175 (7.9)	4.34 (4.16–4.53)	9.83 (8.45–11.37)	2,039,835	1,869,150	2,741,427	188
Men	186 (10.7)	147 (8.5)	69 (4.0)	7.37 (7.08–7.66)	5.62 (4.47–6.97)	996,024	891,920	1,412,042	107
Foot pain									
Women	380 (17.2)	288 (13.0)	141 (6.4)	4.63 (4.42–4.83)	8.30 (7.04–9.73)	1,863,311	1,542,450	2,504,188	172
Men	164 (9.4)	147 (8.5)	58 (3.3)	6.25 (5.96–6.54)	6.41 (5.18–7.85)	878,215	891,920	1,245,026	94

^a^ The re-occurrence risk ratio presents the ratio of the pain risk/probability at the second follow-up among those who reported the same pain type at the first assessment to the outcome probability among those who did not report the pain at the first follow-up.

^b^ The incidence rate is the number of new cases at the second follow-up by the total number of person-years, expressed per 1,000 person-years.

^c^ The predicted UK population estimates were obtained using 1996 pain episode, and 2004 and 2012 pain when walking estimates and applied to 2004, 2012, and 2030 UK population. According to the ONS, there were 9,967,191 women older than 50 years in 1989 and 10,139,958 in 1996 in the United Kingdom. In the same age group, there were 10,851,333 women and 9,296,226 men in 2004, and 11,852,232 women and 10,533,146 men in 2012. The 2020-based estimates for the UK 2030 population indicate that there will be 14,583,601 women and 13,179,059 men older than 50 years.

^d^ The prevalence of musculoskeletal pain in 2030 in the United Kingdom presented as number (rounded to an integer) of cases per 1,000 individuals.

^e^ In the Chingford study, the presented estimates of pain episodes were based on 587 women aged 50 years and older. There were no reports on hip and foot pain in 1989.

**Figure 2:**
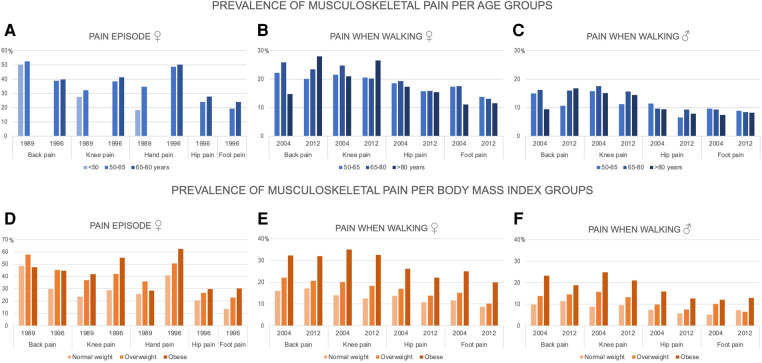
The prevalence of musculoskeletal pain per age and body mass index groups. (**A–C**) The prevalence of musculoskeletal pain episodes in women (the Chingford study) and often troubled by pain when walking in women and men (the ELSA study) per age groups (<50, 50–65, 65–80, ≥80 years) in 1989, 1996, 2004, and 2012; (**D−F**) The prevalence of musculoskeletal pain episode in women (the Chingford study) and often troubled by pain when walking in women and men (the ELSA study) per body mass index groups (normal weight: 18.5–25 kg/m^2^, overweight: 25–30 kg/m^2^, and obese: ≥30 kg/m^2^) in 1989, 1996, 2004, and 2012; the percentages of underweight (<18.5 kg/m^2^) participants were <1%, and the estimates were not shown in this group.

Figure 2 shows the prevalence of musculoskeletal pain per age and BMI group. We did not find a consistent age trend among older adults. Interestingly, hand pain episodes almost doubled in 50–65-year-old women compared with younger than 50 years. However, we did observe that the prevalence of musculoskeletal pain increased with higher BMI. Further, we found that using a BMI cut-off of 27 kg/m^2^ (Table 3), the prevalence risk ratio of knee pain among women who had BMI of ≥27 kg/m^2^ was from 1.49 (95% CI 1.29–1.70) for pain episodes to 2.49 (95% CI 2.31–2.67) higher for pain when walking than among women with BMI of <27 kg/m^2^.

**Table 3:** Musculoskeletal pain prevalence and prevalence ratio among individuals with a body mass index above 27 kg/m^2^.

	Pain episode					
	1989			1996		
	Prevalence		Prevalence ratio	Prevalence		Prevalence ratio
	*N* (%)		(95% CI)	*N* (%)		(95% CI)
	<27 kg/m^2^	≥27 kg/m^2^		<27 kg/m^2^	≥27 kg/m^2^	
Back pain						
Women	300 (50.1)	132 (56.2)	1.12 (0.98–1.26)	174 (35.5)	152 (44.2)	1.24 (1.08–1.41)
Knee pain						
Women	162 (27.0)	95 (40.4)	1.49 (1.29–1.70)	160 (32.7)	168 (48.8)	1.50 (1.33–1.66)
Hand pain						
Women	170 (28.4)	78 (33.2)	1.17 (0.95–1.39)	219 (44.7)	191 (55.5)	1.24 (1.11–1.38)
Hip pain						
Women	—	—	—	106 (21.6)	103 (29.9)	1.38 (1.15–1.62)
Foot pain						
Women	—	—	—	82 (16.7)	91 (26.5)	1.58 (1.32–1.85)
	Pain when walking					
	2004			2012		
	Prevalence		Prevalence ratio	Prevalence		Prevalence ratio
	*N* (%)		(95% CI)^a^	*N* (%)		(95% CI)^a^
	<27 kg/m^2^	≥27 kg/m^2^		<27 kg/m^2^	≥27 kg/m^2^	
Back pain						
Women	191 (17.8)	326 (28.5)	1.60 (1.44–1.76)	176 (17.5)	344 (28.5)	1.89 (1.73–2.05)
Men	85 (10.7)	180 (19.1)	1.79 (1.55–2.03)	94 (12.4)	165 (16.9)	1.36 (1.13–1.60)
Knee pain						
Women	157 (14.7)	347 (30.4)	2.07 (1.90–2.24)	136 (13.5)	337 (28.0)	2.49 (2.31–2.67)
Men	84 (10.6)	201 (21.4)	2.03 (1.79–2.26)	77 (10.2)	174 (17.8)	1.75 (1.50–2.00)
Hip pain						
Women	148 (13.8)	268 (23.5)	1.70 (1.52–1.88)	114 (11.3)	235 (19.5)	1.90 (1.70–2.11)
Men	62 (7.8)	124 (13.2)	1.69 (1.40–1.98)	46 (6.1)	101 (10.3)	1.70 (1.37–2.04)
Foot pain						
Women	130 (12.1)	250 (21.9)	1.80 (1.61–2.00)	96 (9.5)	192 (15.9)	1.80 (1.57–2.03)
Men	53 (6.7)	111 (11.8)	1.77 (1.46–2.09)	45 (5.9)	102 (10.4)	1.76 (1.42–2.09)

^a^ The prevalence (risk) ratio presents the ratio of the pain risk/probability among those who had a body mass index of ≥27 kg/m^2^ to the pain probability among those who had a body mass index of <27 kg/m^2^ at the same assessment.

## Discussion

We found that musculoskeletal pain was prevalent among UK individuals aged 50 years and older, and back and knee pain were the most prevalent. Prevalence and incidence rates were higher in women than men. However, men had higher 8-year re-occurrence risks of all musculoskeletal pain types than women, indicating their tendency to report more persistent pain. The experience of musculoskeletal pain episodes was associated with an approximately two times higher risk but being often troubled by pain when walking indicated four to seven times higher risk of the same pain in 8 years. We did not observe prevalence trends per age group, except for hand pain that peaked in 50–65-year-old women. Yet, we found an increasing trend per BMI group and supportive results for the BMI of 27 kg/m^2^ cut-off in musculoskeletal research. In 2030, we expect 2–7 million people aged 50 years and older in the United Kingdom to seek site-specific musculoskeletal pain-focused healthcare.

Our study used high-quality UK population-representative cohorts, included five musculoskeletal sites, first time provided the incidence and re-occurrence risk, considered sex, ageing, and obesity impacts, and predicted national estimates for 2030. However, we acknowledge several limitations. Firstly, the 8-year participation of the same individuals in the ELSA study was lower than that in the Chingford study. At the first set of follow-ups, we had a quarter of the Chingford women younger than 50 years and 3.4% of the ELSA participants older than 80 years. In longitudinal studies, the older participants tend to drop off earlier due to morbidity or mortality over time (15). In addition, the ELSA waves were not focused on following the same individuals, allowing different household members to be interviewed and including new participants over time. We showed the estimates per age group to minimise the differences, but estimates for the participants older than 80 years from the ELSA first follow-up are unlikely to be population-representative. Secondly, the pain episode estimates were unavailable for men because of the study design. Yet, we could compensate for that lack with the other cohort and report important sex differences. Also, the back pain episode estimates might not be the 8-year. The participants experiencing the condition of interest when asked are more likely to recall similar past events; however, we cannot rule out whether that back pain happened 2 or more years ago. Thirdly, we did not consider the underlying disease that caused the musculoskeletal pain. The most common disease is osteoarthritis (OA), and frequent troubled pain when walking is likely OA pain. The relationship between structural changes and pain is inconsistent, and we focused on the pain that the patients are concerned with the most and leads to healthcare expenditures (5, 26). Finally, we did not consider the effect of treatment that could affect pain reports. Our outcome was binary that minimises the misclassification. Pain severity would be affected by analgesics and orthopaedic procedures, but given that these treatments are not fully successful, the binary outcome should be the least affected by treatment effects (27).

Formulations of pain questions and study samples vary across studies influencing the estimates, comparisons, and interpretations. Studies using different questions in the same population are needed to help bridge and optimally interpret the evidence for public health and policy matter. The evidence is increasing that musculoskeletal pain is a huge burden, negatively affecting the ability to work and quality of life (4, 9, 28–33). The estimates in the older but still working population are essential because sick leave, reduced productivity, and early retirement contribute to socioeconomic burden (34). In the United Kingdom, the current state pension age is 66 years but enounced to increase to 68 years (35). We showed here that musculoskeletal pain is a considerable problem in UK individuals aged 50–65 years, i.e., before retirement. A US study assessed several types of musculoskeletal pain, and found, similarly to us, that the most prevalent in older adults were back and knee pain (4). We also showed that hip and foot pain were similarly prevalent, and the prevalence did not change much with the stringency of pain questions, indicating that hip and foot pain might manifest in more severe forms. It is of clinical and public health importance highlighting the need for carefully choosing treatments and considering that these patients will require a longer or repeated use of pain-focused healthcare. Interestingly, the hand pain prevalence is rarely reported, and two studies reported estimates similar with ours from 1989, but significantly lower than ours from 1996 (4, 10). Importantly, we observed that in women, hand pain episodes doubled in age 50–65 years compared with the age below 50 years and remained similar in age 65–80 years.

For the first time, we provided musculoskeletal pain incidence and re-occurrence risk using two questions and two cohorts from the same population. In our cohorts, the data collection was done in the late 1990s and the early 2000s. Although the prevalence changes, it is essential to have the estimates over different periods from high-quality studies to be able to observe the change, design public health interventions, and assess the effects of these interventions. We used different pain questions and aimed to provide a nexus between them. As expected, we found that a looser definition such as “any pain episode” resulted in higher prevalence and incidence and lower 8-year re-occurrence than a definition closer to chronic pain focused on quality of life instead of duration. The re-occurrence risk accounts for re-occurrence and persistent conditions. Importantly, we found that men had a higher re-occurrence risk of all musculoskeletal pain types than women. Thus, men reported pain less often, but in a more persistent/severe form.

Further, we observed increasing prevalence trends per BMI group in older adults. Therefore, future changes in musculoskeletal pain prevalence and incidence could change because of population lifestyle and health programs targeting obesity. The predicted 2030 estimates considered sex and age increase but not BMI, so if obesity increases/decreases, our prediction will underestimate/overestimate the reality. Notably, the average 8-year BMI difference in the United Kingdom was larger in the late 1990s (Chingford) than early 2000s (ELSA). Compliance with and attrition from weight management programs in older individuals is challenging, and engagement alternatives are needed. In a 19-year follow-up study, musculoskeletal pain and obesity were bidirectionally related and should be considered together in public policies (15). Also, a BMI of 25–27 kg/m^2^ was not associated with pain or mortality (15). Therefore, advising older patients with musculoskeletal pain to attain a BMI of <27 kg/m^2^ could be an alternative, relaxed goal. It could reduce the long-term risk of musculoskeletal pain while at the same time keeping other health risks low (15). Here, we confirmed that women and men with BMI of ≥27 kg/m^2^ were almost twice more likely to experience any musculoskeletal pain than those with BMI <27 kg/m^2^.

## Conclusions

Musculoskeletal pain was highly prevalent in UK population-representative cohorts, thus, presenting a significant healthcare problem. Women had higher prevalence and incidence of musculoskeletal pain than men, but men reported more persistent pain. Back and knee pain were the most common, and the prevalence varied on the stringency of pain questions. Hip and foot pain were less reported, but different questions did not significantly change the prevalence. Hand pain peaked in women aged 50–65 years. As the population ages, more individuals will seek pain-focused healthcare, but future changes in the prevalence and incidence are likely to change with BMI. For older adults, a BMI of 27 kg/m^2^ should be further explored and considered as a relaxed goal for weight management, as it could reduce the musculoskeletal pain risk and, consequently, pain-focused healthcare expenditures.

## Data Availability

The data analysed in this study are subject to the following licenses/restrictions: requests to access the Chingford 1000 Women Study data should be addressed to julie.damnjanovic@ndorms.ox.ac.uk. All research proposals requesting data access will need specification of the analysis plan and approval of the scientific board before any data can be released. Data from the English Longitudinal Study of Ageing are available at the UK Data Service upon user registration.
